# Greenhouse Gas Inventory Model for Biochar Additions to Soil

**DOI:** 10.1021/acs.est.1c02425

**Published:** 2021-10-12

**Authors:** Dominic Woolf, Johannes Lehmann, Stephen Ogle, Ayaka W. Kishimoto-Mo, Brian McConkey, Jeffrey Baldock

**Affiliations:** † School of Integrative Plant Sciences, Cornell University, Ithaca, New York 14953, United States; ‡ Cornell Atkinson Center for Sustainability, Cornell University, Ithaca New York 14953, United States; § Natural Resource Ecology Laboratory, Colorado State University, Fort Collins, Colorado 80523, United States; ∥ Institute for Agro-Environmental Sciences, National Agriculture and Food Research Organization, Ibaraki, 305-8604, Japan; ⊥ Ministry of Agriculture and Agri-Food, Ottawa K1A 0C5, Canada; # CSIRO—Commonwealth Scientific and Industrial Research Organisation, Glen Osmond 5064 Australia

**Keywords:** climate-change mitigation, carbon sequestration, carbon-dioxide removal, soil carbon

## Abstract

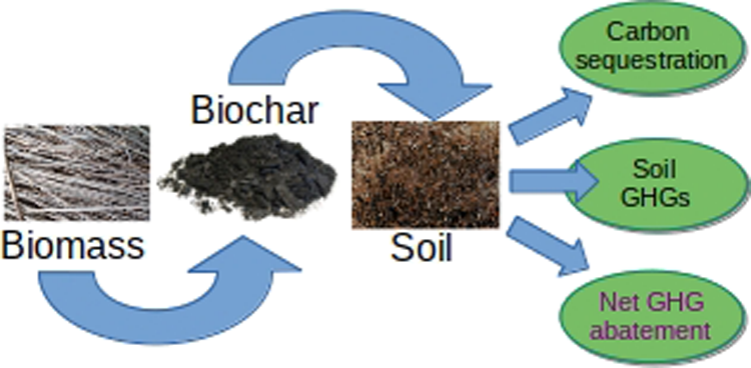

Stabilizing the global climate within safe bounds will require greenhouse gas (GHG) emissions to reach net zero within a few decades. Achieving this is expected to require removal of CO_2_ from the atmosphere to offset some hard-to-eliminate emissions. There is, therefore, a clear need for GHG accounting protocols that quantify the mitigation impact of CO_2_ removal practices, such as biochar sequestration, that have the potential to be deployed at scale. Here, we have developed a GHG accounting methodology for biochar application to mineral soils using simple parameterizations and readily accessible activity data that can be applied at a range of scales including farm, supply chain, national, or global. The method is grounded in a comprehensive analysis of current empirical data, making it a robust method that can be used for many applications including national inventories and voluntary and compliance carbon markets, among others. We show that the carbon content of biochar varies with feedstock and production conditions from as low as 7% (gasification of biosolids) to 79% (pyrolysis of wood at above 600 °C). Of this initial carbon, 63–82% will remain unmineralized in soil after 100 years at the global mean annual cropland-temperature of 14.9 °C. With this method, researchers and managers can address the long-term sequestration of C through biochar that is blended with soils through assessments such as GHG inventories and life cycle analyses.

## Abstract 2

In this article, we develop a simple and robust empirical model of the avoided greenhouse gas (GHG) emissions due to addition of biochar to mineral soils, suitable for use in GHG inventories or life-cycle assessments.

## 1 Introduction

Biochar is the pyrogenic carbon-rich solid formed through pyrolysis of a biomass feedstock (i.e., heating it in an anaerobic environment). Biochar production, together with its storage in soil, has been proposed as a means to mitigate climate change by sequestering carbon in a more persistent form than the raw biomass from which it is generated,^1−3^ thus lowering the rate at which photosynthetically fixed C is returned to the atmosphere.^4^ The net impact of a system in which plants fix atmospheric carbon dioxide (CO_2_) through photosynthesis, with some of that fixed carbon then sequestered in biochar before it can be returned to atmospheric CO_2_ through respiration or combustion, is to remove CO_2_ from the atmosphere. Most climate-change mitigation scenarios now recognize that maintaining a safe climate will require CO_2_ removal (CDR), most critically to offset hard to eliminate emissions and potentially also to recover from an overshoot in safe CO_2_ concentration.^5^ Biochar production represents one of the few established methods to viably provide CDR at a scale large enough to substantially mitigate climate change.^6^

Given the rising imperative for and interest in CDR, there is a clear need for methods to quantify the greenhouse gas (GHG) impact of biochar production and sequestration at a range of scales from farm to national inventory. In particular, many applications will require GHG accounting methods that can be conducted with relatively simple and accessible input data. For example, National Inventories may lack detailed information about the soils or cropping practices in which biochar is applied, or projects in developing countries may lack the capacity to conduct chemical analyses of the biochar. Although some previous studies estimate the carbon sequestration or GHG mitigation impacts of biochar addition to soils, none fully address these requirements. Life-cycle assessments (LCAs), for example, relate only to specific conversions of specific feedstocks applied in specific locations and are not generalizable.^7−9^ A recent meta-analysis of biochar impacts on soil organic carbon (SOC) quantifies only the short-term sequestration, without accounting for long-term decay dynamics or other GHG impacts, and does not disaggregate by different feedstocks or production conditions.^10^ Estimates of global or regional climate-change mitigation potential have relied on simplified estimates of biochar permanence that do not account for carbon-sequestration variability due to feedstock, production method, biochar chemical composition, or climate at the site of application.^3,11,12^ Some initial progress toward establishing GHG accounting protocols for biochar has been developed in the gray literature, such as an assessment of analytical methods to determine biochar carbon stability^13^ and a voluntary carbon market protocol for biochar which indicates that a model must be used to determine biochar persistence but does not itself provide such a model.^14^

An important milestone in the establishment of GHG accounting protocols for biochar addition to soils is the biochar guidance developed by the Intergovernmental Panel on Climate Change (IPCC) as a basis for future methodological development for UNFCCC signatory countries to quantify their annual GHG sources and sinks.^15^ Nonetheless, a widely applicable general method to estimate carbon sequestration in agricultural soils from application of various biochars remains a gap in the literature. The IPCC guidance method focused only on national-scale accounting using pyrolysis production conditions as a sole criteria for quantifying biochar persistence because pyrolysis temperature can be easily monitored. It does not provide a method to estimate biochar persistence from the chemical properties of the substance nor does it account for variability in persistence due to climate at the site of sequestration. Although the guidance was included in the 2019 refinement report updates to the IPCC GHG Guidelines,^15^ it was only as an annex that is not part of good practice for national GHG inventories, because of the IPCC requirement that all methodologies have existing support in the literature.

The IPCC guidelines comprise three tiers. Tier 1, the simplest, consists of linear equations that relate activity data to their resultant GHG fluxes using default coefficients known as emission factors (EFs). Tier 1 is intended for application in all countries with a minimum amount of activity data. Tier 2 methods are based on similar equations, but with countries providing their own nationally or regionally specific EFs. Tier 3 methods can involve the application of detailed dynamic models that may require subject expertise to use. The IPCC does not stipulate which models must be used at tier 3 but provides guidance on good practice for the application of models. Although it is good practice to use country-specific tier 2 or tier 3 methods, many countries, particularly in the developing world, rely on tier 1 methods. Accordingly, the method described in Ogle et al.^15^ was designed to be a simple EF-based approach that can be applied by any country wishing to report the effect of land application of biochar sequestration on GHG fluxes. Beyond national inventories, EF-based models are widely used in a variety of contexts including voluntary and compliance reporting of GHG mitigation, LCAs, policy, and project planning at scales ranging from farm to global.

In this article, we further develop the IPCC biochar method, providing expanded analysis of the scientific background, updated EFs based on more recent data, and new parameterizations that can reduce uncertainty when users have access to more detailed information such as elemental composition of the biochar.

## 2 Materials and Methods

### 2.1 Scope of the Biochar GHG Model

#### 2.1.1 Thermochemical Conversion Technologies

Carbonized pyrogenic organic matter (pyOM) is generated when biomass undergoes incomplete combustion or heating under anaerobic conditions. However, not all such materials are sufficiently persistent to provide a viable means of carbon sequestration. In particular, pyOM generated at low temperatures or under moist conditions shows short residence times in the soil environment. Torrefaction and hydrothermal carbonization typically utilize temperatures below 350 °C, with torrefaction operating under dry feedstock conditions in ambient pressure while hydrothermal carbonization uses pressurized aqueous slurries. Torrefaction and hydrothermal carbonization are excluded from this methodology because they do not generate solid products that are significantly more persistent in soil than the original organic feedstock.^16,17^

Processes included in this methodology that utilize higher temperature thermochemical conversion of dry materials can be classified as either pyrolysis (in which oxidants are excluded) or gasification (in which oxidant concentrations are low enough to generate syngas).^18^

#### 2.1.2 GHG Sources and Sinks Associated with Biochar

The climate-change mitigation potential of adding biochar to soils depends largely on the quantity of biomass carbon sequestered in the biochar and the rate at which it is returned to the atmosphere.^4^ More readily decomposed un-pyrolyzed biomass will rapidly return most of its carbon to the atmosphere if subject to fire or allowed to decompose.^19^ Therefore, the un-mineralized carbon stocks remaining are larger for biochar than for raw biomass that would have otherwise decomposed or burned, once the cumulative mineralization from biomass decay exceeds that from pyrolysis and biochar decomposition.^19^ Provided that sustainably sourced feedstocks are utilized which do not entail large production emissions or a reduction in forest cover, this persistence-derived carbon sequestration is typically the largest influence of biochar on net GHG balances, although other GHG fluxes can also be significantly influenced by biochar amendment to soils.^3,7,20−22^ Other GHG impacts arising from the full life-cycle of biochar production and application include the following:

1. Modification (typically a reduction) of N_2_O^23^ and CH_4_^24^ emissions from soil.
2. Conversion of biomass to biochar can avoid emissions of N_2_O and CH_4_ that would have arisen from the decomposition or combustion of that biomass.^3^
3. Biochar can increase net primary productivity,^25,26^ thereby increasing net removal rates of atmospheric CO_2_, particularly if the increased biomass is itself utilized for carbon sequestration or bioenergy.^3^
4. Biochar can alter the decomposition rate of native SOC, an interaction referred to as “priming”, thereby increasing or decreasing non-pyrogenic SOC stocks.^27−29^
5. Reduced fertilizer requirements from improved nutrient-use efficiency can reduce GHGs from fertilizer production and transport.^3^
6. Co-production of bioenergy with biochar can offset fossil-fuel emissions.^22,30,31^
7. Approximately 50% of biomass-carbon is emitted as volatile and gaseous organic compounds during pyrolysis.^32^ A well-engineered modern pyrolysis plant ensures that organic compounds are fully combusted to CO_2_.^22,33^ However, simple low-cost technologies may not fully combust these and may emit CH_4_ and volatile organic compounds^33−35^ together with other GHGs derived from sulfur or nitrogen in the biomass.^36^
8. Loss of carbon stocks in vegetation could occur if unsustainable biomass supply chains are adopted, particularly if woody biomass from trees that would otherwise have remained alive is utilized or if there were deforestation to convert land into biomass plantations.^3^
9. Finally, there may be emissions associated with growing and transport of biomass feedstocks, particularly if dedicated biomass crops are used rather than wastes or residues.^37^

Many GHG inventory methodologies take a sectoral approach in which GHG sources and sinks are reported by the economic sector. The IPCC (2019) GHG guidelines,^15^ for example, provide separate methodologies for energy; industrial processes and product use; Agriculture, Forestry and Other Land Use (AFOLU); and waste. Within each sector, there are a variety of source categories associated with specific emission and removals such as changes in soil organic C stocks or N_2_O emissions from agricultural soil management. The biochar methodology presented here focuses on the direct effect of biochar amendments on SOC stocks and GHG fluxes in soils.

A full LCA of the greenhouse impacts of biochar systems would also include all the indirect sources and sinks itemized above. For example, in national GHG inventories reporting to the UNFCCC, methods are already provided in the IPCC guidelines to account for the other fluxes. Specifically, GHG fluxes associated with growing biomass feedstocks (including land use change, if any) would be estimated using the land use sections in Volume 4 (Agriculture, Forestry and Other Land Use; “AFOLU”) of the IPCC guidelines. Changes in GHG emissions arising from diversion of waste streams into biochar feedstocks would be estimated with methods in the waste sector (Volume V in IPCC 2019). For plant residues and manures, their utilization as biochar feedstock could reduce the input of this organic material to soil and thereby can lower soil C stocks in croplands and grasslands and possibly other land uses receiving manure amendments, with such changes addressed with other methods in the SOC section of the IPCC guidelines (Volume 4 in IPCC 2019). Emissions during manufacture of biochar should be reported in either the energy or industrial sectors (depending on whether there is energy co-production with the biochar). Emissions from fuel use for transport of feedstock and biochar and for agricultural operations to incorporate biochar would also be included in the energy sector.

For application of the methods for purposes other than national inventories, the emission effects from biochar application will depend on the boundaries of the system. For example, if biochar production is a co-product of energy production, then the emission for the production of biochar can be attributed to that energy production and only the emissions after production attributed to emission effects of land application.

#### 2.1.3 Carbon Sequestration in Biochar

The amount of carbon sequestered depends on the quantity of biochar added to soil, the carbon fraction of the biochar, and the fraction of its carbon that is mineralized to CO_2_ over a given time period. The first of these parameters, the amount of biochar generated and added to soil, must be tracked as an input to this GHG inventory method. To ensure that the method can readily be applied across a variety of conditions and circumstances, alternative parameterizations are provided that allow biochar’s carbon fraction and persistence to be estimated either from its production method or from chemical analyses of its organic carbon and hydrogen content, depending on which type of data are available. The organic carbon fraction of biochar can be estimated from the production method (pyrolysis or gasification) and feedstock (Section 2.2). The decomposition rate of biochar may be estimated from the pyrolysis temperature or from its elemental composition (the ratio of hydrogen to organic carbon, H/C_org_, specifically) if these data are available (Section 2.3). Thus, inventory compilers need only collect activity data on the quantity of biochar added to soil, the type of feedstock from which it was produced, the temperature of pyrolysis, and optionally H/C_org_. Where neither pyrolysis temperature or H/C_org_ are available, the methodology can be applied to approximate conservative estimates of the biochar persistence based on the values derived for low-temperature biochar, which is less persistent than biochar produced from the same material at higher temperatures (see Section 2.3).

### 2.2 Carbon Fraction of Biochar

If the biochar organic carbon content (*F*_C_, defined as the organic carbon mass fraction on a dry mass, DM, basis) has been measured directly, this value may be used in the following methodology. Otherwise, *F*_C_ can be estimated according to the following method. The organic carbon content of biochar on a dry ash-free (DAF) basis (*F*_C,daf_) can be approximated by an exponential regression function (eq 1) of pyrolysis temperature (*T* in °C) from a meta-analysis^38^ with 128 measurements from 26 papers (*R*^2^ = 0.65).
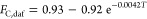
1

To avoid the need to measure ash content, we require the organic carbon fraction of the biochar on a DM basis. The ash content of the biochar (*F*_a,bc_) is related to the biomass ash (*F*_a,bm_) by eq 2, assuming that ash is conserved during pyrolysis.
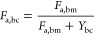
2where *Y*_bc_ is the yield of DAF biochar from pyrolysis, expressed as a fraction of DAF biomass feedstock.

The DAF biochar yield, *Y*_bc_, is calculated as a function of feedstock lignin content (*L*) and pyrolysis temperature (*T*) using eq 3 from another meta-analysis^39^ (*n* = 146 from 18 articles, *R*^2^ = 0.65).
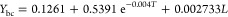
3

The carbon fraction of the biochar on a DM basis (*F*_C_) is then given by eq 4.
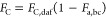
4Data on the ash (*n* = 1276) and lignin (*n* = 516) content of biomass feedstocks, which are parameters in these regression equations, were taken from the Phyllis2 database for biomass, algae, feedstocks for biogas production, and biochar^40^ and are summarized in Table 1. The biomass types included in Table 1 represent a range of the most globally abundant feedstocks. If the biochar GHG method is to be applied to feedstocks other than those included in Table 1 (such as food waste, which is too variable in composition to represent accurately with an average value), then the carbon content of the resultant biochar (*F*_C_) would need to be measured directly.

**Table 1:** Ash, Carbon (C), and Lignin Content for Different Classes of Biomass^a^

feedstock	ash	sd	*n*		C	sd	*n*		lignin	sd	*n*
bagasse	5.8	4.4	20		49.0	2.2	18		17.7	2.7	13
bamboo	3.9	2.6	6		48.3	1.2	6		23.3	2.1	6
herbaceous	7.0	5.4	495		48.9	2.4	390		11.8	7.1	294
maize stover	5.2	3.9	29		47.7	2.4	18		9.5	4.8	28
manure	28.5	15.2	30		45.2	9.9	36		11.3	11.3	5
paper sludge	32.7	14.1	16		48.4	9.2	12		23.5	6.4	4
pits/shells/stones	3.8	3.2	119		50.6	4.3	119		33.2	14.8	53
rice residues	17.9	3.9	42		48.3	3.2	33		17.9	9.6	13
sewage sludge	39.4	9.9	54		51.1	5.6	56		6.0	9.7	13
wheat straw	7.2	3.8	104		48.8	1.5	69		12.3	7.0	42
wood	2.2	3.9	507		50.7	2.1	488		24.7	6.8	136

a Ash and lignin mass fractions are given on a DM basis. Carbon is given on a DAF basis. Rice residues include both rice hulls and rice straw. Herbaceous feedstocks include grasses, forbs and leaves, excluding rice husks and straw. Values provided are the means, number of samples (*n*), and standard deviations (sd) of data provided in the Phyllis2 database of biomass and waste (ECN, 2021).

### 2.3 Permanence

The permanence of a carbon stock relates to the longevity of the stock, that is, how long the increased carbon stock remains in the soil or vegetation.^41^ This is linked to consideration of the reversibility of the increased carbon stock. Biochar typically decomposes at least 1–2 orders of magnitude more slowly in soil than the biomass from which it was made.^4^ This increased persistence is attributed to condensation reactions that generate fused aromatic molecular structures during pyrolysis,^42−44^ which are less readily decomposed by microbial activity.^45^ The degree of condensation and aromaticity of biochar increases with increasing pyrolysis temperature and with increasing pyrolysis reaction time.^44,46^

Biochar is a complex mixture of both aliphatic and aromatic organic compounds, with the larger aromatic structures typically being more persistent than the other compounds. As such, biochar decomposition is best described using a multi-pool decay function rather than a single pool model. At least a two-pool exponential decay model, comprising fast- and slow-mineralizing fractions, is required to extrapolate decay over centennial timescales.^47^ Accordingly, we applied a minimum of a two-pool exponential decay model fitted to published biochar decomposition data. For studies where a two-pool model was not a good fit, a three-pool model was used instead. The data used to develop the model were derived through a comprehensive literature survey, subsequently filtered to include only those studies that provided a minimum of 1 year of decay data to which a multi-pool model could be fitted.

GHG inventories require consistency for reporting the GHG impact of all activities, which typically demands a single representative value for the mitigation impact of an activity. Because biochar sequestration is a dynamic process in which the stored carbon mineralizes gradually over long time periods, representation of this dynamic process as a single value is achieved by defining a representative time period over which to integrate the GHG impact. This is analogous to global warming potentials (GWPs) for non-CO_2_ gases—defined as the time-integrated radiative forcing due to a pulse emission of a given component relative to a pulse emission of an equal mass of CO_2_—which, because of their differing persistence in the atmosphere, also vary depending on the time period over which the GWP is integrated. The UNFCCC has adopted a 100-year period as the basis for calculating GWPs for national inventories and mitigation targets, which balances the need to be both sufficiently long to recognize the cumulative impact of GHG concentrations on long-term climate stabilization while also being sufficiently short to measure the progress toward mitigation targets over the coming years and decades.

For this biochar method, we do not specify the time period to use but provide estimates of biochar permanence over a range of representative timescales from 100 to 1000 years. The spreadsheet provided as the Supporting Information also provides the functionality to calculate permanence factors for any desired timeframe. We do, nonetheless, recommend adoption of a 100-year period for similar reasons to those outlined above for GWPs. Using a period shorter than 100 years would provide a biased overestimate of the mitigation impact from biochar over the century timescales that are highly policy-relevant. However, a longer time would underestimate the impact over the coming century that is the main focus of the current climate policy. It was also for these reasons that a 100-year permanence metric for biochar was suggested in the IPCC 2019 guidelines. Although adopting a 100-year time frame means that the gradual emissions from biochar decomposition after this time are not accounted for, this can be resolved in future inventories when it becomes necessary to do so. If biochar were to form a substantial component of mitigation efforts over the coming century, then future inventory systems in the 22nd century and onward would need to recognize the ongoing emissions from biochar decay as a net CO_2_ source.

The decomposition data in the studies used were measured at a range of soil temperatures. These were recalculated to a common basis for the mean annual soil temperature at the site of application, assuming that Q_10_ varies with temperature (*T*) according to *Q*_10_ = 1.1 + 12.0 e^–0.19*T*^, after Lehmann et al. (2015).^47^ The mean annual temperature of the world’s croplands is 14.9 °C, derived as a spatial mean of WorldClim 2.1 data^48^ over the global distribution of cropland.^49^

### 2.4 Other GHG Fluxes from Soils

Effects of biochar additions on the exchange of the GHGs methane (CH_4_), nitrous oxide (N_2_O), and CO_2_ between soil and atmosphere were derived by a review of published literature, with an emphasis on quantitative systematic reviews and meta-analyses. Where the effect of biochar was not significantly different from zero at *p* < 0.05, no effect was assumed. The results of these meta-analyses and their implementation in the biochar GHG model are provided in Section 3.3.

## 3 Results and Discussion

### 3.1 Modeled Carbon Fraction of Biochar

The biochar carbon fractions (*F*_C_) for different classes of biochar, summarized by feedstock type and production conditions, are shown in Table 2. The three representative temperatures provided in Table 2 may be used to designate biochar into temperature classes of low (pyrolyzed at between 350 and 450 °C), medium (450–600 °C), and high (≥600 °C). The sensitivity of *F*_C_ to pyrolysis temperature is low, particularly for feedstocks with a higher ash content (Table 2). This is because the increase in carbon concentration in the organic fraction of the biochar at higher pyrolysis temperatures is somewhat compensated by the increasing concentration of ash that also correlates with higher temperatures. Due to this low temperature sensitivity, the simplification was made in the IPCC 2019 guidelines^15^ to use only a single representative value of *F*_C_ for each feedstock based on the average of the three representative temperature ranges. We also provide values of *F*_C_ that are averaged over pyrolysis temperature in Table 2. However, it should be noted that when the pyrolysis temperature is known, for example when it is already provided as an input parameter for the persistence calculations (Section 3.2 below), using the temperature-dependent values of *F*_C_ will provide greater accuracy without incurring greater data-collection demands.

**Table 2:** Carbon Content (*F*_C_) for Different Classes of Biochar, Summarized by Feedstock Type and Production Conditions^a^

	*F*_C_ as a function of pyrolysis temperature				
feedstock	low	medium	high	mean	gasification
bagasse	0.57 (0.16)	0.62 (0.18)	0.64 (0.20)	0.61 (0.18)	0.43 (0.11)
bamboo	0.66 (0.04)	0.72 (0.05)	0.75 (0.06)	0.71 (0.05)	0.51 (0.14)
herbaceous	0.60 (0.09)	0.65 (0.11)	0.66 (0.13)	0.64 (0.11)	0.38 (0.10)
maize stover	0.63 (0.06)	0.68 (0.08)	0.70 (0.09)	0.67 (0.08)	0.45 (0.12)
manure	0.39 (0.09)	0.39 (0.11)	0.39 (0.11)	0.39 (0.10)	0.14 (0.04)
paper sludge	0.39 (0.26)	0.41 (0.29)	0.42 (0.31)	0.41 (0.29)	0.12 (0.03)
pits/shells/stones	0.67 (0.06)	0.73 (0.08)	0.76 (0.09)	0.72 (0.08)	0.52 (0.14)
rice residues	0.46 (0.05)	0.48 (0.06)	0.48 (0.07)	0.47 (0.06)	0.20 (0.05)
sewage sludge	0.35 (0.25)	0.37 (0.28)	0.38 (0.29)	0.37 (0.27)	0.10 (0.03)
wheat straw	0.59 (0.06)	0.64 (0.08)	0.65 (0.09)	0.63 (0.08)	0.38 (0.10)
wood	0.70 (0.05)	0.77 (0.06)	0.81 (0.07)	0.76 (0.06)	0.63 (0.17)

a Rice residues include both rice hulls and rice straw. Herbaceous feedstocks include grasses, forbs, and leaves, excluding rice husks and straw. Production conditions are aggregated into classes of gasification or pyrolysis at low (350–450 °C), medium (450–600 °C), or high (≥600 °C) temperatures. Biochar pyrolysis temperatures below 350 °C were excluded. The average carbon fraction on a DM basis (*F*_C_) is given as the mean over all three temperature ranges for biochar produced through pyrolysis and as a separate value for gasification biochar. sd are shown in parentheses.

The carbon fraction of biochar produced from gasification is also shown in Table 2 for materials in which the ash residue has not been separated from the organic carbon component. In this case, *F*_C_ of gasification biochar can be as low as 0.1–0.14 for high-ash feedstocks such as sewage sludge or manure. Such gasification-derived residues with high ash to organic carbon ratios may alternatively be described as “ash with biochar” or as “high-ash content biochar”, with no established standard specifying which term is to be preferred. Nonetheless, because this methodology only considers sequestration of the organic carbon fraction of the material, the presence of additional ash (which can sometimes also provide further agronomic benefits in terms of nutrient supply and pH regulation) does not affect the calculation of carbon sequestration. For gasification-derived biochar in which the ash component has been fully or partially removed, the *F*_C_ values in Table 2 should not be used, and the carbon content should instead be measured directly.

### 3.2 Modeled Permanence of Carbon Removals

**Figure 1:**
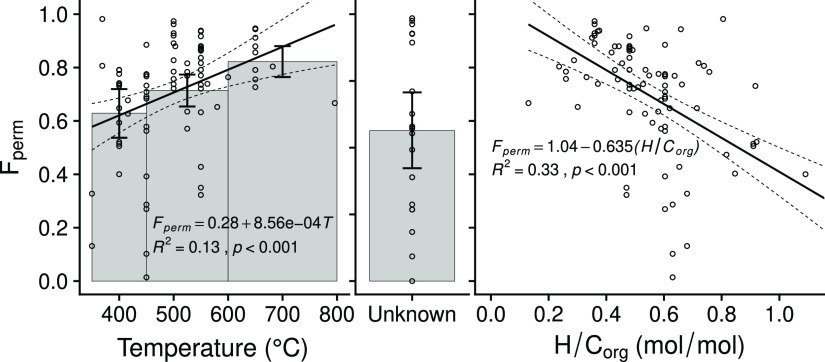
Fraction of biochar carbon remaining in soil after 100 years (*F*_perm_) as a function of pyrolysis temperature (left panel) and biochar molar hydrogen to organic carbon ratio (H/C_org_; right panel). The center panel shows data where neither pyrolysis temperature nor H/C_org_ are known and where physical movement cannot be distinguished from mineralization (hence persistence is underestimated). In addition to a linear regression against pyrolysis temperature, the left panel indicates mean values for low (350 ≤ *T* < 450 °C), medium (450 ≤ *T* < 600° C)), and high (*T* ≥ 600 °C) pyrolysis-temperature classes. Biochar pyrolysis temperatures below 350 °C were excluded. In all cases, *F*_perm_ values were calculated for a soil temperature of 14.9 °C (the mean annual air temperature in croplands globally). Error bars and dashed lines indicate 95% confidence intervals.

Figure 1 shows the fraction of biochar carbon remaining (*F*_perm_) after 100 years at the global mean annual cropland temperature, both as a function of pyrolysis temperature and as a function of biochar H/C_org_. Figure 1 (center panel) also shows the 100-year *F*_perm_ for published studies where the pyrolysis temperature was unknown (typically from natural pyrogenic carbon production during wildfires), with the mean carbon fraction remaining under these uncontrolled conditions being 56% of the initial pyrogenic carbon that remains after the wildfire has passed. The biochar mineralization studies and their respective fitted decay models are shown in the Supporting Information.

Two alternative parameterizations of biochar permanence are provided here as alternative metrics to use in a GHG inventory. The first parameterization uses pyrolysis temperature to estimate permanence, and the other uses the molar hydrogen to organic carbon ratio (H/C_org_) of the biochar. H/C_org_ is a proxy for the degree of condensation of the material because the larger the aromatic structures become, the more C to C bonds they form at the expense of H–C bonds. Organic oxygen to organic carbon ratios would provide a similar proxy for condensation, but organic oxygen is more difficult to analytically distinguish from inorganic oxygen in the ash than is the case for hydrogen, which typically is not present in the ash in significant amounts. The use of H/C_org_ rather than O/C_org_ thus becomes especially important when considering biochar with a high ash content such as that produced during gasification and/or derived from ash-rich feedstocks. Gasification biochars are typically produced at higher temperatures than pyrolysis, leading to low H/C_org_ ratios that correlate with high persistence.^30,50^

The second form of model parameterization uses pyrolysis temperature, which is also a proxy for condensation. For a given reaction time, higher temperatures increase the condensation of the product. However, temperature is less closely related to condensation because the degree of carbonization also varies with reaction time, among other factors. For this reason, where H/C_org_ values are known or can reasonably be obtained, it is recommended that they are used as the basis for permanence estimation rather than pyrolysis temperature, which has a lower correlation with persistence (Figure 1). However, in many situations, access to laboratories and equipment to conduct elemental analysis may be limited, in which case, production conditions would be the most appropriate parameterization.

For the pyrolysis-temperature parameterization, the fraction of biochar carbon (*F*_perm_) that remains after a given time *T* was derived by binning empirical measurements of biochar permanence (derived using a multi-pool exponential decay model as described in Materials and Methods above) into representative pyrolysis temperature ranges of low (350 ≤ *t* < 450 °C), medium (450 ≤ *t* < 600 °C), and high (*t* ≥ 600 °C). The means and standard errors of *F*_perm_ in each temperature range are provided in Table 3 for representative periods of time ranging from 100 to 1000 years and soil temperatures from 5 to 25 °C.

**Table 3:** Permanence Coefficients for Biochar Carbon Sequestration as a Function of Soil Temperature and Time^a^

		*F*_perm_ as function of pyrolysis temperature			H/C_org_ regression coefficients		
soil temperature (°C)	time (years)	low	medium	high	*c*_hc_	*m*_hc_	*R*^2^
5.0	100	0.84 (0.037)	0.89 (0.018)	0.94 (0.0086)	1.13	–0.46	0.31
10.0	100	0.72 (0.042)	0.79 (0.026)	0.88 (0.019)	1.10	–0.59	0.33
15.0	100	0.63 (0.045)	0.71 (0.03)	0.82 (0.028)	1.04	–0.64	0.32
20.0	100	0.57 (0.047)	0.67 (0.032)	0.79 (0.033)	1.01	–0.65	0.31
25.0	100	0.54 (0.049)	0.64 (0.033)	0.76 (0.037)	0.98	–0.66	0.30
10.9	100	0.7 (0.042)	0.77 (0.027)	0.87 (0.021)	1.09	–0.60	0.33
14.9	100	0.63 (0.045)	0.71 (0.03)	0.82 (0.028)	1.04	–0.64	0.32
5.0	500	0.55 (0.048)	0.66 (0.032)	0.78 (0.037)	0.99	–0.65	0.30
10.0	500	0.3 (0.052)	0.44 (0.035)	0.57 (0.061)	0.74	–0.60	0.23
15.0	500	0.19 (0.05)	0.32 (0.033)	0.44 (0.071)	0.57	–0.49	0.17
20.0	500	0.15 (0.049)	0.26 (0.031)	0.37 (0.074)	0.48	–0.43	0.13
25.0	500	0.13 (0.049)	0.23 (0.03)	0.34 (0.075)	0.43	–0.39	0.12
10.9	500	0.27 (0.052)	0.41 (0.035)	0.54 (0.064)	0.71	–0.58	0.21
14.9	500	0.19 (0.05)	0.32 (0.033)	0.44 (0.071)	0.57	–0.50	0.17
5.0	1000	0.35 (0.051)	0.49 (0.034)	0.63 (0.058)	0.80	–0.62	0.24
10.0	1000	0.14 (0.049)	0.25 (0.031)	0.37 (0.075)	0.47	–0.42	0.13
15.0	1000	0.083 (0.048)	0.16 (0.026)	0.25 (0.073)	0.30	–0.27	0.07
20.0	1000	0.066 (0.047)	0.12 (0.023)	0.2 (0.069)	0.23	–0.21	0.04
25.0	1000	0.06 (0.047)	0.1 (0.021)	0.17 (0.066)	0.20	–0.17	0.03
10.9	1000	0.12 (0.049)	0.23 (0.03)	0.34 (0.075)	0.43	–0.38	0.12
14.9	1000	0.084 (0.048)	0.16 (0.026)	0.25 (0.073)	0.30	–0.28	0.07

a Soil temperatures are provided in 5 °C increments from 5 to 25 °C and also at the mean annual temperatures of global croplands (14.9 °C) and US croplands (10.9 °C). The fraction of biochar carbon remaining (*F*_perm_) is shown for pyrolysis temperature ranges of low (350–450 °C), medium (450–600 °C), and high (≥600 °C), with standard errors in parentheses. Linear regression coefficients of *F*_perm_ against H/C_org_ are also given in the right three columns for use in eq 5.

For the H/C_org_ parameterization, *F*_perm_ was expressed as a linear regression against H/C_org_ (eq 5). The regression coefficients (intercept = *c*_hc_, slope = *m*_hc_, and *R*^2^) for this equation are shown in Table 3 for representative time periods ranging from 100 to 1000 years and soil temperatures from 5 to 25 °C. These linear regressions were significant at *p* < 0.001 for all time periods and soil temperatures.
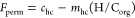
5Where it is neither possible to measure H/C_org_ nor to obtain reliable data on production conditions, a conservative estimate of biochar permanence could be adopted using the value of *F*_perm_ derived for low-temperature pyrolysis (Table 3). For general use, it is suggested to use *F*_perm_ values calculated at a soil temperature of 14.9 °C, the global average. For more accurate regionally specific calculations, the local mean annual temperature should be used either from the values in Table 3 or using the spreadsheet provided as the Supporting Information, which allows recalculation of permanence values at any soil temperature.

### 3.3 Other GHG Fluxes from Soils

#### 3.3.1 Priming

Biochar can affect the turnover rate of existing non-pyrogenic SOC. Such changes in mineralization are referred to as priming. The standard convention is that increases in mineralization are referred to as positive priming and the converse being negative priming.^29,51^ Several papers have summarized biochar effects on SOC priming.^4,27,52,53^ A recent meta-analysis of 21 studies reported a 4% mean decrease in SOC mineralization with biochar additions,^4^ although the 95% confidence interval included zero. All published experiments available for inclusion in this meta-analysis consisted of two-component studies with biochar and SOC interactions quantified but without any new plant-root or plant-biomass C added. Modeling,^54^ long-term incubations,^55,56^ and long-term field studies^57,58^ all support the expectation that priming will become increasingly negative rather than positive over a period of several years. However, given that the net negative priming was not significant at *p* < 0.05 in currently available meta-analyses, priming is conservatively not included in this GHG accounting methodology. As more data become available to better constrain the long-term impacts of priming, biochar GHG methodologies could address this either by incorporating the impacts of biochar into dynamic SOC models^54^ where these are applied in the methodology or as a multiplicative correction factor on long-term (steady-state) SOC stocks where these are used as the basis for SOC accounting (as, e.g., in IPCC (2019) tier 1).

It should be noted that the meta-analysis results indicated in the previous paragraph apply only to mineral soils. Few studies have investigated priming by biochar in organic soils or forest soils with substantial organic horizons. One study on priming of forest soil organic horizons found substantial losses of carbon over a ten-year period with charcoal additions.^59^ However, this study was unable to attribute these losses to the organic soil carbon or to the charcoal because isotopic labeling was not employed.^60^ Nor could carbon mineralization be separated from leaching of dissolved or colloidal organic carbon that can be stabilized in the underlying mineral soil.^60^ Nonetheless, the GHG accounting methodology presented here should, conservatively, not be applied in organic (i.e., Histosols) or forest soils having an organic horizon due to the possibility of positive priming.

#### 3.3.2 Nitrous Oxide

Biochar can alter nitrous oxide emissions from soil, typically reducing emissions. Meta-analyses using inverse variance weighting indicated a mean reduction in N_2_O emissions of 54% (*n* = 261, 30 studies),^23^ 12.4% (*n* = 122, 40 studies),^61^ and 38% (*n* = 435, 48 studies).^62^ However, using inverse variance weighting in the meta-analysis of biochar priming has been questioned because it does not account for non-independence of treatments within studies. Re-analyzing their same data set, weighted by the inverse of the number of observations per site to account for non-independence, Verhoeven et al.^61^ found that this reduced the effect size to 9.2% which was not significant at *p* < 0.05. The persistence of nitrous oxide reductions over time is also important to quantifying the net impact of biochar on emissions. The observed impact of biochar on nitrous oxide emissions has tended to be reduced over time and has not been unambiguously demonstrated as statistically significant after 1 year^62^ in part due to the smaller effect size over time but also due to a paucity of long-term data. For the method described here, we accordingly include only N_2_O impacts during the first year after biochar amendment and only for biochar additions in excess of 10 Mg C ha^–1^. To derive an estimate of effect size, we used the data set provided by Borchard et al.,^62^ filtered to exclude pot trials or incubations which are typically not representative of field conditions. The field-trial data included the crops rice (*n* = 37), vegetable (28), maize (9), other cereals (12), perennial crops (6), and other crops (9). Nitrogen fertilizer type in the field trials was reported as urea (*n* = 47), none (16), other mineral (21), mixed mineral and organic (14), and organic (3). The filtered data were then analyzed using robust variance estimations^63^ with random effects, including study as a random effect. This gave an effect size of a 23% reduction in N_2_O emissions (95% confidence interval of 5–41% reduction). Note that this effect on N_2_O emissions is not specific to the source of N, such as fertilizer, mineralization of plant litter or soil organic matter, and so on but rather is a percent reduction for the entire flux of direct N_2_O emissions from the land parcel.

Although this biochar GHG model includes the above methodology for quantifying N_2_O impacts, we recognize that the GHG impact of a 1-year reduction in N_2_O emissions is typically small relative to the carbon sequestration. For this reason, inclusion of N_2_O impacts in the overall GHG balance should be regarded as optional since their omission would have little effect on the final result.

#### 3.3.3 Methane

Many studies have shown changes in methane fluxes to or from soils in response to biochar application.^64−68^ However, there is little consensus on which conditions correspond to increased or decreased net emissions or uptake. For example one meta-analysis found that biochar increased CH_4_ emissions from paddy soils by a mean of 19%,^69^ whereas another meta-analysis found that biochar decreased CH_4_ in paddy soils,^24^ and a third meta-analysis concluded that there was negligible change in CH_4_ emissions from paddy soils.^66^ Notwithstanding the lack of consensus about which conditions lead to increases or decreases in emissions, all of these meta-analyses concluded that there was no significant change in CH_4_ across all studies. Accordingly, for this methodology, it was assumed that there are no net changes in methane emissions or uptake in soils.

### 3.4 Overall GHG Inventory Method

The overall method for estimating GHG impacts of biochar additions to mineral soils can thus be summarized using eq 6.
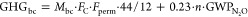
6where GHG_bc_ is the net avoided GHG emissions in units of CO_2_-equivalent (CO_2_e), *M*_bc_ is the mass of biochar added to soil, *F*_C_ is the organic carbon fraction of the biochar, 44/12 is the conversion factor from carbon to CO_2_e, *F*_perm_ is the fraction of biochar organic carbon remaining after a defined period of time (100 years, unless a clear rationale is provided to use a different time period), *n* is the baseline annual nitrous oxide emissions (i.e., the emissions without biochar) from the total area of land over which biochar is applied at an application rate in excess of 10 Mg C ha^–1^, and GWP_N2O_ is the GWP of nitrous oxide. It is recommended to use the most recent available IPCC value (currently, this is 273 for a 100-year GWP^70^).

When *F*_C_ has been measured for the specific biochar being applied, this value can be used directly in eq 6. Otherwise, *F*_C_ is estimated as a function of feedstock and production method using Table 2. When biochar H/C_org_ has been measured for the specific biochar being applied, *F*_perm_ should be derived using eq 5 together with the coefficient values provided in Table 3. If H/C_org_ is not available, then *F*_perm_ should be estimated from pyrolysis temperature class in Table 3. If neither H/C_org_ nor pyrolysis temperature are known, then a conservative estimate of *F*_perm_ should be applied using the values in Table 3 corresponding to low-temperature pyrolysis.

#### 3.4.1 Worked Example

A brief worked example is provided here to demonstrate how this inventory method is applied. For example, we consider conversion of maize stover to biochar through pyrolysis with a maximum temperature during thermochemical conversion of 500 °C. For this example, we assume that biochar C fraction and H/C_org_ have not been measured directly. The conversion temperature of 500 °C lies in the medium range of pyrolysis temperatures. Thus, from Table 2, *F*_C_ is equal to 0.68. Assuming that a 100-year permanence timeframe is applied and the biochar is sequestered in cropland soil with a mean annual temperature of 10 °C, then from Table 3, we find that *F*_perm_ is equal to 0.79. For the nitrous oxide impact, we assume that 15,000 Mg of biochar is added to 1000 ha of cropland, which receives 150 kg of mineral nitrogen fertilizer ha^–1^ yr^–1^ but no leguminous or organic matter nitrogen additions. Using the IPCC tier 1 method to estimate annual N_2_O emissions from the cropland,^15^ total annual N_2_O emitted from land receiving greater than 10 Mg ha^–1^ of biochar is given by 1000 ha × 0.15 Mg N ha^–1^ × 0.01 Mg N_2_O–N Mg^–1^ N × 1.57 Mg N_2_O Mg^–1^ N_2_O–N = 2.4 Mg N_2_O. Now, using eq 6 and assuming a GWP_N_2_O_ of 298, net avoided GHG emissions are equal to 15,000 Mg of biochar × 0.68 × 0.79 ×44/12 + 2.4 × 298 = 29,710 Mg CO_2_e.

### 3.5 Context of Biochar within an Overall GHG Accounting Framework

We have noted that given the rising urgency of finding ways to remove excess CO_2_ from the atmosphere, there is a clear need for GHG accounting protocols that quantify the mitigation impact of CDR practices, such as biochar, that have the potential to be deployed at scale. Many situations demand GHG accounting methodologies that can be conducted using limited amounts of data that can readily be acquired and modeling tools that are simple enough to apply without high levels of technical expertise. Here, we have developed such a GHG accounting methodology for biochar application to mineral soils that could feasibly be applied at farm, supply chain, national, or global scales using activity data that can be broadly available. Despite its simplicity, it is grounded in a comprehensive analysis of current empirical data, making it a robust method that has potential to form a basis for, for example, national inventories and voluntary and compliance carbon markets, among other applications. This robustness is enhanced by the decision to exclude any avoided GHGs that are not yet determined to be statistically significant in meta-analyses, even where such impacts are supported by mechanistic understanding of the processes.

When implementing biochar application as a carbon credit scheme, an LCA of pyrolysis-biochar-soil system may also be required. For example, when Japan registered “biochar addition to mineral soil in cropland/grassland” as a new methodology based on the IPCC (2019) model in the National GHG Credit scheme (J-Credit), a relevant LCA GHG estimate was needed (https://japancredit.go.jp/english/methodologies). An LCA can compare the associated GHGs from the overall system (including biomass provision and biochar production) to soil application as many studies have been conducted.^7,71−75^ As described in Section 2.1.2, LCA boundaries need to be considered to meet the purpose of the target biochar scheme.

Future improvements to GHG accounting for biochar soil amendments will require a focus on a few key areas of research where data and mechanistic studies are still sparse. Notably in this regard, although reductions in N_2_O emissions from soil are now unambiguously demonstrated during the first year after biochar application, too few long-term field studies and too little mechanistic understanding stymie our ability to make robust predictions of N_2_O impacts into the longer term. Although changes in CH_4_ fluxes from soils have been frequently reported in response to biochar, there are currently too few empirical data and too little mechanistic understanding to predict reliably the size or direction of this effect under different field conditions. Although long-term incubations, studies of soils with historical accumulations of pyrogenic carbon, and modeling all support the view that negative priming of non-pyrogenic SOC (npSOC) will tend to increase npSOC stocks in the long term, more long-term studies are still required to better constrain the size of this effect, and incorporation of biochar priming interactions into dynamic SOC models will also be needed for better prediction of the this effect over time. Finally, more data on biochar decomposition will better constrain the interactions between permanence and environmental covariates of decomposition (such as soil texture and moisture) and biochar properties such as its ash composition.

Despite these ongoing needs for further research, the current methodology by virtue of making conservative assumptions about each of these impacts can already be used with confidence that it will not be overestimating the mitigation impact of biochar additions.

## Supporting Information Available

Data used to derive the permanence factors for biochar together with their respective references; functionality to recalculate the fraction of biochar remaining after decomposition (*F*_perm_) for different time periods and for different soil temperatures; and the software code (written in R) required to calculate the effect size for biochar impacts on N_2_O emissions (XLSX)
